# Perceptions and attitudes of medical students toward AI in anatomy learning

**DOI:** 10.1186/s12909-026-10085-1

**Published:** 2026-08-03

**Authors:** Ibrahim Hoja, Anas J. Mistareehi, Wafaa Mahmoud, Dana Abumadi, Mohammad Isied, Heba Ghozlan, Ayman Mustafa, Mohammed Z. Allouh

**Affiliations:** 1 Department of Anatomy, Faculty of Medicine, Jordan University of Science and Technology, Irbid, Jordan; 2 Faculty of Medicine, Jordan University of Science and Technology, Irbid, Jordan; 3 Department of Physiology and Biochemistry, Faculty of Medicine, University of Science and Technology, Irbid, Jordan; 4 Department of Basic Medical Sciences, College of Medicine, QU Health, Qatar University, Doha, Qatar; 5 Department of Anatomy, College of Medicine and Health Sciences, United Arab Emirates University, Al Ain, United Arab Emirates

**Keywords:** Artificial intelligence, ChatGPT, Anatomy education, Medical students, Perceptions, Attitudes, Medical curriculum, AI literacy

## Abstract

**Background:**

Artificial intelligence (AI) tools are increasingly being integrated into medical education. However, their use in anatomy learning, particularly among medical students in the Middle East, remains underexplored.

**Objective:**

To assess medical students’ perceptions, attitudes, and patterns toward AI use for anatomy learning, and to examine associations with sex, academic level, and GPA.

**Methods:**

A cross-sectional study was conducted among medical students at Jordan University of Science and Technology. A validated 36-item questionnaire (Cronbach’s α = 0.785–0.867) was distributed online. Responses were analyzed using descriptive and inferential statistics, including chi-square tests and Spearman’s rank correlation, to identify factors associated with AI use and perceptions of AI in anatomy learning.

**Results:**

Among 351 respondents, 300 (85.5%) use AI for anatomy, with 95.7% of them using it during exam preparation. ChatGPT was the dominant platform (96.0%). Gross anatomy was the most studied topic. Students strongly endorsed AI platforms for understanding concepts, immediate answers, 24/7 availability, and time-saving. Major concerns included a lack of visual/lab experience, premium costs, and AI misunderstanding questions. Overall, among the AI-using subgroup, 75.3% of students supported the formal integration of AI, but 52.3% believed AI could replace aspects of traditional teaching. Two gender-related patterns were observed in the unadjusted analyses, but neither remained statistically significant after FDR correction. Higher academic performance was associated with lower agreement that AI could replace traditional teaching or function as a standalone learning resource.

**Conclusions:**

Medical students use AI as an anatomy learning adjunct, valuing its conceptual and accessibility benefits while recognizing its inability to replace hands‑on dissection. Higher GPA was only associated with lower agreement that AI could serve as a complete standalone resource for anatomy learning. These findings support structured AI literacy training, equitable access, and balanced curricula AI integration that preserves hands-on foundations.

**Supplementary Information:**

The online version contains supplementary material available at https://doi.org/10.1186/s12909-026-10085-1.

## Introduction

Human anatomy remains one of the foundational basic sciences in undergraduate medical education, as understanding it is essential for safe clinical practice. Anatomy teaching has undergone substantial transformation over the last few decades, driven by curricular integration, expanding biomedical content, and other factors such as resource constraints [1, 2]. Traditional anatomy education has relied on didactic lectures, cadaveric dissection, and atlas-based learning. Although cadaver-based instruction still holds substantial educational value, its implementation has become increasingly constrained [1, 3]. Many medical schools have documented decreases in anatomy contact hours and shifted toward integrated curricula that rely increasingly on multimodal teaching resources [1, 4], while ongoing ethical, logistical, and infrastructural challenges continue to complicate anatomy education globally [5]. Digital technologies such as three-dimensional visualization, virtual dissection tables, virtual reality applications, and online interactive resources have become progressively embedded in anatomy teaching. However, no single instructional modality suffices for all curricular aims in anatomy pedagogy. A multimodal approach integrating complementary tools based on learner needs could offer strong potential for artificial intelligence (AI) as a transformative educational technology [6, 7].

AI has expanded rapidly across health professions education. In medical education, AI applications have been explored for adaptive tutoring, automated feedback, personalized learning pathways, content generation, assessment support, and simulation-based training [8, 9]. There is increasing interest in using AI in anatomy learning because of its potential to enhance visualization, enable interactive question-answering, and support self-directed revision outside regular classroom hours [10, 11]. A recent systematic review found that AI tools may improve engagement, personalization, and anatomical understanding, especially when integrated with other digital learning resources [11]. Similarly, a study in clinical anatomical education has emphasized that AI may extend human teaching capacity and offer novel educational affordances, but only when implemented cautiously and with attention to the specific complexities of the discipline [10]. Different studies have shown that many students perceive AI as useful, innovative, and likely to become an integral part of future medical practice; however, these same studies also consistently highlight major gaps in formal training and curriculum exposure [12–14].

Educational accuracy is essential in anatomy learning. Unlike general domains, AI systems must handle precise terminology, spatial relationships, normal variation, and clinical detail [10]. While AI enhances accessibility and interactivity, it introduces risks including factual inaccuracies, fabricated content, bias, incomplete explanations, and overreliance [8, 10, 15]. Moreover, AI may struggle with anatomical variation, uncertainty, and the tacit knowledge gained through hands-on teaching, while concerns regarding hallucinations, ethical issues, reduced critical thinking, and the need to verify AI-generated information remain important educational challenges [8–10].

Despite growing literature on AI in medical education, limited research has specifically explored medical students’ perceptions of AI as a tool for anatomy learning. In Jordanian medical schools, where increasing educational technology intersects with high-demand anatomy teaching, understanding students’ use of these platforms may support more evidence-based educational decisions. This study aimed to assess medical students’ perceptions and attitudes toward the use of AI in anatomy learning, including patterns of use, perceived benefits and limitations, trust and verification practices, and perspectives on integrating AI into anatomy curricula, while offering insight into the evolving role of AI in anatomy education.

## Methods

### Study design and participants

This was a cross-sectional study conducted among second- and third-year medical students at Jordan University of Science and Technology (JUST). These academic years were specifically selected because students are actively engaged in anatomy courses and are therefore familiar with the subject matter relevant to this study.

At JUST, medical students are admitted through three tracks, regular, parallel, and international, which differ by admission competitiveness and student origin. Jordanian students enter through regular and parallel programs. The international track admits non-Jordanian students. Nevertheless, all students follow the same curriculum, teaching methods, and assessments. All second- and third-year medical students at JUST (*N* = 1,302) were invited to participate via three repeated official emails. A total of 351 students completed the survey, corresponding to a response rate of 27.0% (351/1,302). Of these, 300 students who reported using AI for anatomy learning met the study inclusion criteria and were included in the final analyses. The required sample size (n) was calculated using Cochran’s formula for finite populations. The following parameters were used in the sample size calculation: a confidence level of 95% (Z = 1.96), a margin of error of 5% (e = 0.05), an expected proportion of *p* = 0.5, and a finite target population of N. Because the target population was finite (*N* = 1,302), the finite population correction (FPC) was applied:

$$\begin{aligned}n=\frac{\frac{Z^{2}\cdot\:p\cdot\:(1-p)}{e^{2}}}{1+\frac{\left(\frac{Z^{2}\cdot\:p\cdot\:(1-p)}{e^{2}}-1\right)}{N}}&=\frac{\frac{(1.96)^{2}\cdot 0.5 \cdot 0.5}{(0.05)^{2}}}{1+\frac{\left(\frac{(1.96)^{2}\cdot{0}.5\cdot{0}.5}{(0.05)^{2}}-1\right)}{1302}}\\&= \frac{384.16}{1+\frac{383.16}{1302}}\\&=296.8\end{aligned}$$

Thus, the required sample size was 297 participants.

### Ethical approval

Ethical approval was obtained from the Institutional Review Board (IRB) at Jordan University of Science and Technology, Irbid, Jordan (Ref. No. Dec2025/188 − 21), non-funded research project Grant No. 20,250,574, before data collection.

### Questionnaire development

The questionnaire was developed in English by two anatomy experts, both of whom had extensive experience teaching human anatomy to medical students. To establish content validity, the draft questionnaire was reviewed by three additional anatomy experts specializing in medical education. These experts evaluated each item for clarity, relevance to the intended constructs, comprehensiveness of content, and appropriateness of phrasing. Their recommendations were systematically incorporated to enhance the quality and conceptual alignment of the survey items. A pilot test with cognitive interviewing using a convenience sample of 10 medical students was conducted to verify that the questionnaire was understandable and relevant to the target population and to improve its clarity and practical applicability. Each student individually completed the survey, provided detailed feedback, and participated in a brief, structured interview designed to identify ambiguous or unclear items. Following the cognitive interview pilot, a separate pilot psychometric study was conducted to examine the internal consistency of the questionnaire before full distribution. The questionnaire was administered to a small sample of medical students (*n* = 20). Cronbach’s alpha was calculated for each multi-item Likert-scale section. The results showed acceptable to good internal consistency: perceived benefits (10 items, α = 0.867), challenges and limitations (5 items, α = 0.817), and future perspectives (4 items, α = 0.785). Based on these results, no items were removed or substantially revised, and the questionnaire proceeded to full data collection.

### Questionnaire structure

The final questionnaire comprises 36 questions divided into seven main sections: The first section collects demographic data such as age, gender, academic year, GPA, and enrollment type. The second explores patterns of AI platform usage in anatomy learning, including frequency, preferred platforms, stages of study, topics, and integration with traditional resources. The third section assesses perceived benefits of AI for comprehension, exam preparation, and engagement. The fourth section identifies challenges and limitations, such as a lack of hands-on experience and unclear explanations. The fifth investigates how students verify and trust AI information compared to traditional methods. The sixth section gathers opinions on future use, recommendations for integration, and the potential replacement of traditional teaching by AI. The final section evaluates students’ confidence in using AI, desired improvements, and overall attitudes toward AI in anatomy education. Question formats included multiple-choice questions, Likert-scale items, and select-all-that-apply questions. The questionnaire required approximately 5–10 min to complete. The questionnaire was administered in English, which is the primary language of instruction at JUST. The full questionnaire is available as Supplementary Material 1.

### Data collection tool and procedures

An online survey was created using Google Forms. A consent form was presented at the beginning of the questionnaire, which participants were required to read and acknowledge before proceeding. The consent form detailed the purpose of the study, the voluntary nature of participation, the assurance of anonymous responses, the confidentiality of all collected data, and the right to withdraw at any time without consequences. Participants who agreed selected an “I agree” option to proceed; those who did not consent were directed to exit the survey and were excluded from the study. The survey was open for 12 weeks, from December 17, 2025, to March 10, 2026.

### Statistical analysis

Data were analyzed using SPSS Statistics version 27.0 (IBM Corp., Armonk, NY, USA). Descriptive statistics were used to summarize participants’ demographic characteristics, patterns of AI platform use, perceptions, challenges, and future perspectives regarding AI use in anatomy learning. Categorical variables were presented as frequencies and percentages.

Responses to Likert-scale items were initially collected using a five-point scale (Strongly Agree, Agree, Neutral, Disagree, and Strongly Disagree). For analytical purposes, responses were regrouped into three categories: Agree (Strongly Agree and Agree), Neutral, and Disagree (Disagree and Strongly Disagree).

Internal consistency reliability was assessed using Cronbach’s alpha. Associations between demographic variables (gender and academic year) and questionnaire responses were examined using the chi-square test of independence. Fisher’s exact test was considered when expected cell counts were small. Correlation analyses were performed to evaluate the relationship between GPA and students’ perceptions toward AI platforms in anatomy learning. Because questionnaire responses were ordinal in nature, Spearman’s rank correlation coefficient (rho) was used. To account for multiple comparisons, p-values from the chi-square tests and Spearman correlation analyses were adjusted using the Benjamini–Hochberg false discovery rate (FDR) procedure. Correlation coefficients and corresponding p-values were reported. All statistical tests were two-tailed, and a p-value < 0.05 was considered statistically significant.

## Results

A total of 351 students responded to the questionnaire. Among them, 51 students (14.5%) reported that they did not use AI for studying anatomy and were therefore excluded from the analysis. Consequently, the final study sample included 300 students who used AI in anatomy learning.

The majority of participating students were female (63.0%, *n* = 189), while males represented 37.0% (*n* = 111). As indicated in Table 1, most participants were 20 years old (50.0%, *n* = 150), followed by 19 years old (31.7%, *n* = 95) and 21 years old (13.0%, *n* = 39).

**Table 1:** Demographic and academic characteristics of the participating students (*N* = 300), including gender, age, current academic year, GPA, and enrollment program. Values are presented as frequency (n) and percentage

Variable	Category	*n* (%)
Gender	Male	111 (37.0%)
	Female	189 (63.0%)
Age (years)	18	2 (0.7%)
	19	95 (31.7%)
	20	150 (50.0%)
	21	39 (13.0%)
	22	7 (2.3%)
	23	5 (1.7%)
	> 24	2 (0.7%)
Current Year	2nd year	141 (47.0%)
	3rd year	159 (53.0%)
GPA	Fair	15 (5.0%)
	Good	28 (9.3%)
	Very good	64 (21.3%)
	Excellent	108 (36.0%)
	Distinguished	85 (28.3%)
Enrollment Program	Regular	194 (64.7%)
	Parallel	49 (16.3%)
	International	57 (19.0%)

Regarding academic year, 53.0% (*n* = 159) of participants were third-year students, whereas 47.0% (*n* = 141) were second-year students. Most participants reported excellent GPA scores (36.0%), followed by distinguished (28.3%), very good (21.3%), good (9.3%), and fair (5.0%). Concerning enrollment programs, students were enrolled in the regular program (64.7%), the international program (19.0%) and the parallel program (16.3%).

### Patterns of AI platform use in anatomy learning

**Fig. 1:**
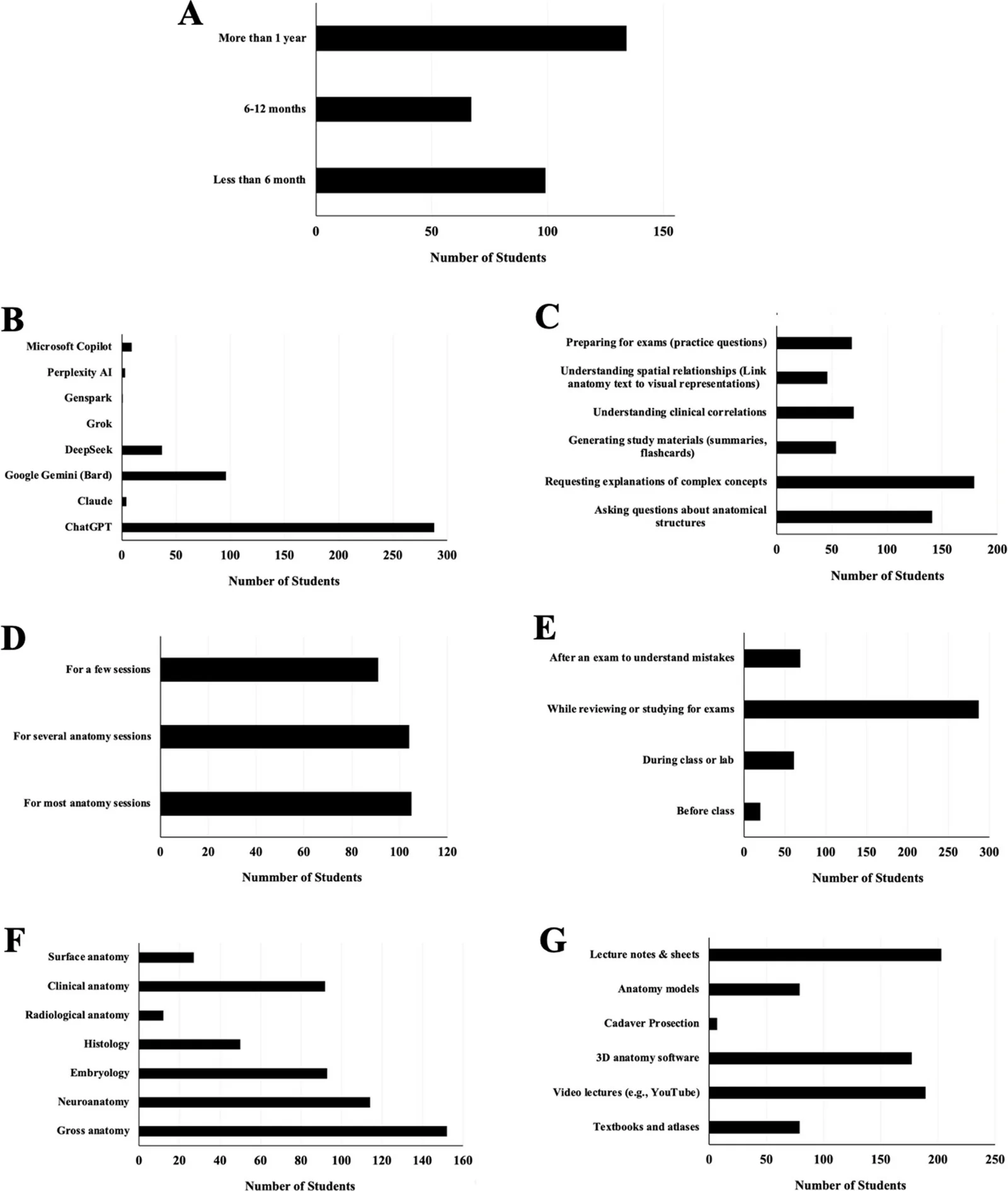
Horizontal bar charts showing patterns of AI platform use among students in anatomy learning. **A** Shows the duration of students’ experience using AI platforms for anatomy learning. **B** Presents the preferred AI platforms used by students. **C** Illustrates the primary purposes for using AI platforms. **D** Demonstrates the frequency of AI platform use during anatomy learning sessions. **E** Shows the situations in which students typically use AI platforms. **F** Presents the anatomical disciplines most studied using AI platforms. **G** Illustrates the traditional learning resources combined with AI platforms. Values are presented as number of students

Our analysis of the AI-using subgroup showed that students widely incorporate AI platforms into anatomy learning and demonstrate substantial use of these tools across different educational contexts. Regarding the length of experience with AI in anatomy learning, nearly half of the participants reported using AI platforms for more than one year (44.7%, *n* = 134), whereas 33.0% (*n* = 99) had used them for less than 6 months and 22.3% (*n* = 67) for 6–12 months (Fig. 1A). Concerning platform preference, ChatGPT was the most commonly used AI platform, reported by 96.0% of participants. Google Gemini represented the second most frequently utilized platform (32.0%), followed by DeepSeek (12.3%). Use of other platforms, including Microsoft Copilot, Claude, Perplexity AI, and Genspark, remained minimal (Fig. 1B).

Regarding primarily using AI platforms for anatomy learning, our survey showed that participants primarily used AI platforms to request explanations of complex anatomical concepts (59.7%, *n* = 179) and to ask questions regarding anatomical structures (47.0%, *n* = 141). Additional applications included understanding clinical correlations (23.3%, *n* = 70), examination preparation through practice questions (22.7%, *n* = 68), generation of study materials (18.0%, *n* = 54), and improving understanding of spatial anatomical relationships (15.3%, *n* = 46) (Fig. 1C). When asked about the frequency of AI platform use in anatomy learning, 35.0% of students reported using AI platforms during most anatomy sessions, while 34.7% indicated using them during several sessions. In contrast, 30.3% of students reported using AI platforms during only a few anatomy sessions (Fig. 1D). When asked about the situations in which they typically use AI for anatomy learning, the vast majority of students reported relying on AI platforms while reviewing or studying for examinations (95.7%, *n* = 287). Smaller proportions indicated using AI during class or laboratory sessions (20.3%, *n* = 61), after examinations to review mistakes (23.0%, *n* = 69), or before classes (6.7%, *n* = 20) (Fig. 1E).

Regarding anatomical disciplines, gross anatomy represented the most common topic studied using AI platforms (*n* = 152), followed by neuroanatomy (*n* = 114), embryology (*n* = 93), and clinical anatomy (*n* = 92). Lower frequencies were reported for histology, surface anatomy, and radiological anatomy (Fig. 1F). When asked which traditional learning resources they combine with AI platforms, students most frequently reported using lecture notes and study sheets (67.7%, *n* = 203), followed by video lectures (63.0%, *n* = 189) and 3D anatomy software (59.0%, *n* = 177). In contrast, cadaver prosection was rarely combined with AI platforms (2.3%, *n* = 7) (Fig. 1G).

### Perceived benefits of AI platforms in anatomy learning

Students evaluated ten statements concerning the benefits of AI platforms in anatomy education using a five-point Likert scale. For analytical purposes, responses were regrouped into three categories: Agree (Agree & Strongly Agree), Neutral, and Disagree (Disagree & Strongly Disagree). The overall pattern indicated consistently favorable perceptions, with agreement levels ranging from 59.0% to 91.3% (Table 2).

**Table 2:** Students’ perceptions of the benefits, challenges, and future perspectives regarding the use of AI platforms in anatomy learning (*N* = 300). Values are presented as frequency and percentage of responses for agree, neutral, and disagree categories

Statement	Agree	Neutral	Disagree
Perceived Benefits of AI Platforms in Anatomy Learning			
AI platforms help me understand and retain anatomical concepts	274 (91.3%)	23 (7.7%)	3 (1.0%)
AI platforms provide immediate answers to my anatomy-related questions	267 (89.0%)	29 (9.7%)	4 (1.3%)
AI offers personalized learning, including native language support	250 (83.3%)	40 (13.3%)	10 (3.3%)
AI platforms are available 24/7, making them convenient	266 (88.7%)	23 (7.7%)	11 (3.7%)
AI platforms help me prepare better for anatomy examinations	250 (83.3%)	41 (13.7%)	9 (3.0%)
AI platforms enhance my critical thinking skills in anatomy	177 (59.0%)	93 (31.0%)	30 (10.0%)
AI platforms help connect anatomical knowledge to clinical applications	233 (77.7%)	56 (18.7%)	11 (3.7%)
AI platforms save time compared to traditional methods	258 (86.0%)	31 (10.3%)	11 (3.7%)
AI platforms make learning more engaging and interesting	202 (67.3%)	69 (23.0%)	29 (9.7%)
AI platforms improved overall academic performance	227 (75.7%)	62 (20.7%)	11 (3.7%)
Challenges and Limitations of AI Platforms in Anatomy Learning			
AI lacks real visual and lab experience	199 (66.3%)	57 (19.0%)	44 (14.7%)
AI platforms sometimes provide complex or unclear explanations	152 (50.7%)	84 (28.0%)	64 (21.3%)
The cost of premium AI platforms is a barrier	186 (62.0%)	68 (22.7%)	46 (15.3%)
AI platforms lack personal interaction and guidance	158 (52.7%)	86 (28.7%)	56 (18.7%)
AI platforms do not always understand user questions	174 (58.0%)	71 (23.7%)	55 (18.3%)
Future perspective			
I plan to continue using AI platforms	276 (92.0%)	18 (6.0%)	6 (2.0%)
I would recommend AI platforms to others	270 (90.0%)	24 (8.0%)	6 (2.0%)
AI platforms should be formally integrated into the curriculum	226 (75.3%)	53 (17.7%)	21 (7.0%)
AI platforms could replace some aspects of traditional teaching	157 (52.3%)	78 (26.0%)	65 (21.7%)

Among students who used AI for anatomy learning, the highest level of agreement was observed for the statement that AI platforms support the understanding and retention of anatomical concepts, with 91.3% (*n* = 274) of students agreeing and only 1.0% (*n* = 3) disagreeing. The immediacy of responses was also highly valued, as 89.0% (*n* = 267) of students agreed that AI platforms provide prompt answers to anatomy-related queries. Similarly, 88.7% (*n* = 266) agreed that AI platforms are available 24/7.

Perceived efficiencies were notable, with 86.0% agreeing that AI tools save time compared to traditional learning methods. Additionally, 83.3% of students agreed that AI supports personalized learning. An identical proportion 83.3% of students agreed that AI platforms facilitate improved preparation for anatomy examinations, while 77.7% (*n* = 233) agreed that AI platforms improve the ability to connect anatomical knowledge with clinical applications.

A moderately high proportion of students (75.7%) agreeing that AI use improved their overall academic performance. Engagement-related benefits were comparatively less pronounced, with 67.3% (*n* = 202) agreeing that AI platforms make learning more engaging and interesting. The statement related to enhancement of critical thinking skills received the lowest level of support, with 59.0% (*n* = 177) of students agreeing.

### Challenges and limitations of AI platforms in anatomy learning

To evaluate potential challenges associated with the use of AI platforms in anatomy education, the students assessed five statements using a five-point Likert scale. For analytical purposes, responses were grouped into Agree (Agree and Strongly Agree), Neutral, and Disagree (Disagree and Strongly Disagree). The distribution of responses is summarized in Table 2. Overall, agreement levels ranged from 50.7% to 66.3%, indicating a moderate acknowledgment of limitations alongside the generally favorable perceptions reported earlier.

The most frequently reported concern was the lack of visual and laboratory experience, with 66.3% (*n* = 199) of students agreeing. Financial constraints also emerged as a notable issue, as 62.0% (*n* = 186) agreed that the cost of premium AI platforms may restrict access for some students.

Despite their utility, AI tools may present barriers related to communication precision and conceptual clarity. More than half of the students identified challenges related to system understanding and communication clarity. Specifically, 58.0% of students agreed that AI platforms do not always understand user questions, while a slightly lower proportion, 50.7%, agreed that AI platforms may provide complex or unclear explanations. Perceptions were comparatively less pronounced regarding the lack of human interaction, although this remained a concern for a slight majority of students (52.7%).

### Future perspectives

Among AI users, students’ views on the future role of AI platforms in anatomy education were assessed using a five-point Likert scale, with responses regrouped into Agree (Agree & Strongly Agree), Neutral, and Disagree (Disagree & Strongly Disagree) categories. The distribution of responses is presented in Table 2.

Our survey analysis indicates a generally positive attitude toward the continued use of AI tools, alongside more cautious attitudes regarding their broader institutional integration and potential to replace traditional teaching methods. Students’ responses generally demonstrated strong acceptance of AI as a supportive and complementary learning tool. A strong majority of students expressed their intention to continue using AI platforms, with 92.0% (*n* = 276) agreeing. Similarly, willingness to recommend AI platforms to peers was notably high, with 90.0% of students agreeing. Support for formal integration of AI platforms into the anatomy curriculum was also substantial, with 75.3% (*n* = 226) of students agreeing. In contrast, perceptions were more divided when considering the potential for AI platforms to replace aspects of traditional anatomy teaching. While just over half of students (52.3%, *n* = 157) agreed with this possibility, a considerable proportion remained neutral (26.0%, *n* = 78) or disagreed (21.7%, *n* = 65).

### Verification and trust in AI platforms for anatomy learning

**Fig. 2:**
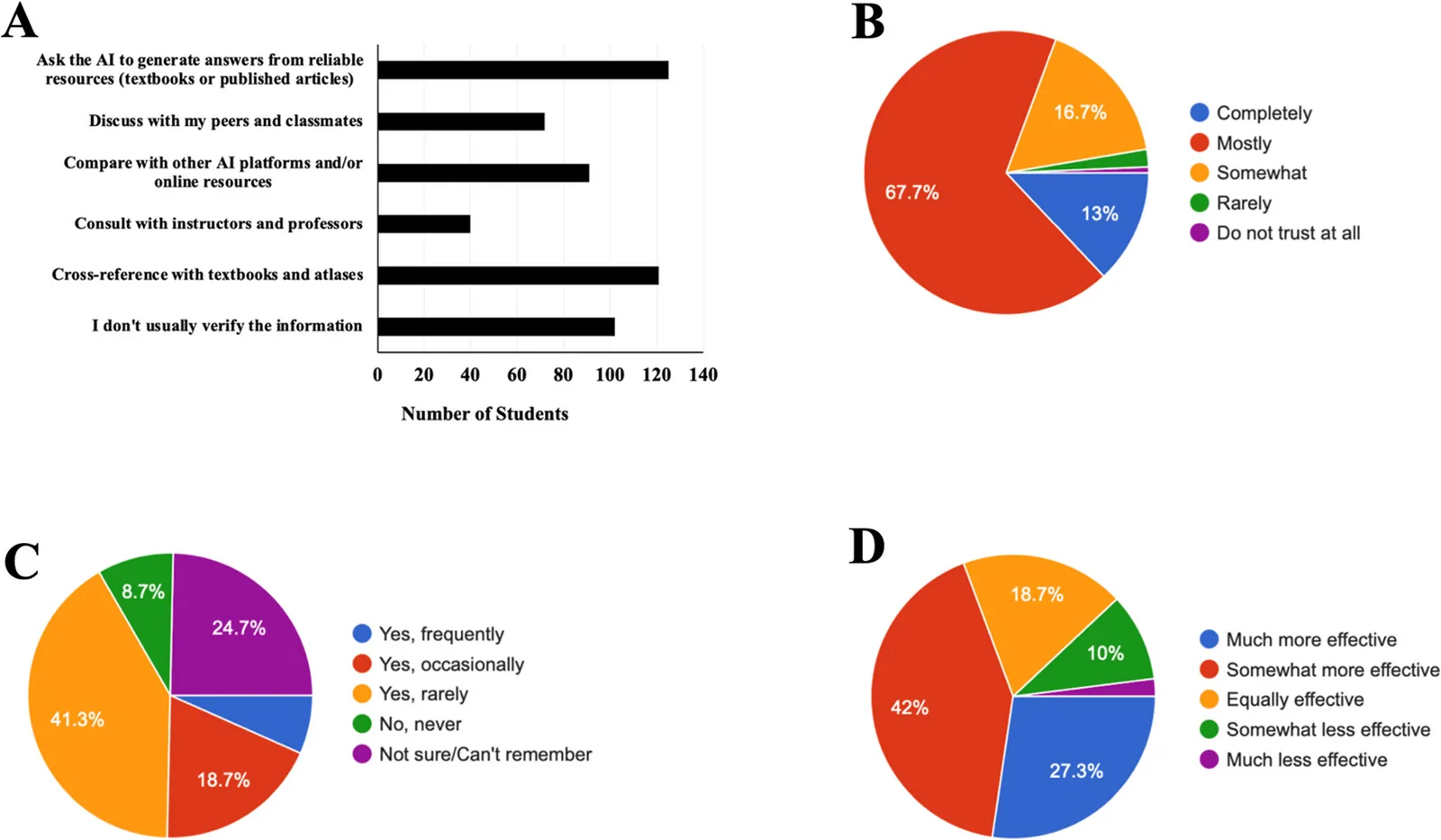
Patterns of verification practices, trust, experiences with inaccurate information, and perceived effectiveness of AI platforms in anatomy learning among students. **A** Horizontal bar chart showing the methods used by students to verify the accuracy of AI-generated information. **B** Pie chart presenting students’ level of trust in AI-generated anatomical information. **C** Pie chart illustrating students’ experiences with receiving incorrect information from AI platforms. **D** Pie chart showing students’ perceptions of the effectiveness of AI platforms compared with traditional anatomy learning methods

Responses regarding verification practices demonstrated variable approaches toward confirming the accuracy of AI-generated information. The most commonly reported strategy was asking AI platforms to generate responses based on reliable sources such as textbooks or published literature (41.7%). Cross-referencing with textbooks and anatomical atlases was also frequently reported (40.3%). A considerable proportion of students compared AI outputs with other AI platforms or online resources (30.3%), while fewer students reported discussing information with peers and classmates (24.0%). Consulting instructors or professors was the least frequently utilized method (13.3%). Notably, 34.0% of students indicated that they do not usually verify the information obtained from AI platforms (Fig. 2A).

Regarding trust, participants generally demonstrated a high level of confidence in AI-generated anatomical information. A majority of students reported either mostly trusting (67.7%, *n* = 203) or completely trusting (13.0%, *n* = 39) such information. Meanwhile, 16.7% (*n* = 50) indicated that they somewhat trust AI outputs. Only a very small proportion expressed low levels of trust, with 2.0% (*n* = 6) reporting that they rarely trust and 0.7% (*n* = 2) indicating that they do not trust AI at all (Fig. 2B).

Experiences with incorrect information varied among students. A substantial proportion of participants reported encountering inaccuracies rarely (41.3%, *n* = 124), while others indicated occasional (18.7%) or frequent (6.7%). In contrast, 8.7% of students reported never receiving incorrect information, while 24.7% were not sure or unable to recall such experiences (Fig. 2C).

When comparing AI platforms with traditional anatomy learning methods, most participants perceived AI as more effective. Specifically, 42.0% (*n* = 126) rated AI as somewhat more effective, and 27.3% (*n* = 82) as much more effective. However, 18.7% (*n* = 56) considered AI to be equally effective as traditional approaches.

### Overall perceptions

**Fig. 3:**
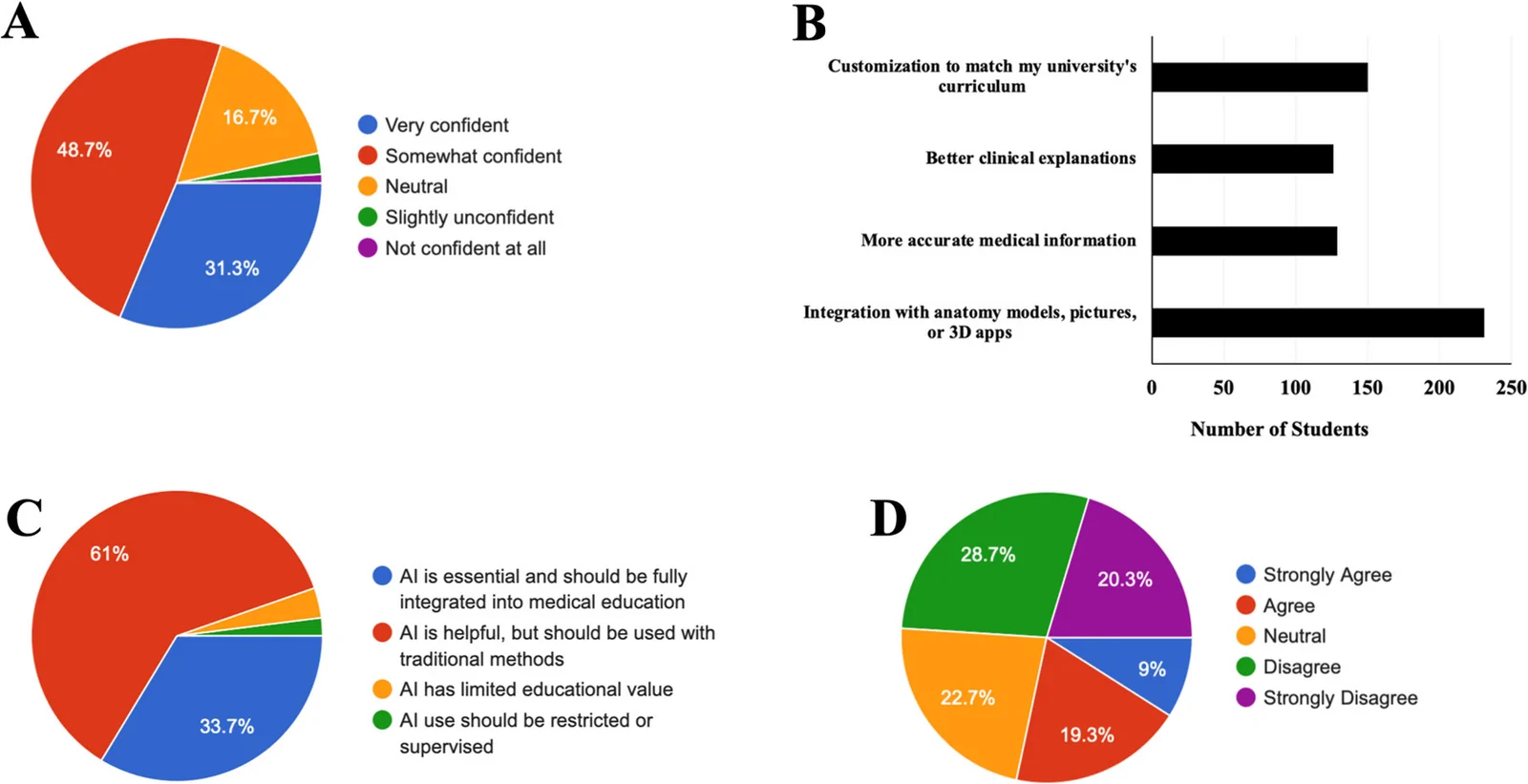
Students’ confidence, perceived improvements, and overall opinions regarding the use of AI platforms in anatomy learning. **A** Pie chart showing students’ confidence in using AI platforms for anatomy learning. **B** Horizontal bar chart illustrating the improvements desired by students for AI platforms, including integration with anatomical models and curriculum customization. **C** Pie chart presenting students’ overall opinions regarding the role of AI in anatomy education. **D** Pie chart showing students’ opinions on using AI platforms as a complete standalone resource for studying anatomy

Students who used AI for anatomy learning reported a high level of confidence in using AI platforms for anatomy learning was observed, and AI tools were generally perceived as valuable complementary educational resources rather than complete replacements for traditional learning methods. Students generally reported a high level of confidence in their ability to use AI tools for anatomy learning. Most respondents described themselves as either very confident (31.3%, *n* = 94) or somewhat confident (48.7%, *n* = 146) (Fig. 3A).

Regarding desired improvements, the most frequently selected feature was integration with anatomical models, images, or 3D applications (77.0%, *n* = 231), highlighting the importance of visual and interactive learning tools. Other commonly identified needs included customization to align with university curricula (50.0%, *n* = 150), more accurate medical information (43.0%, *n* = 129), and improved clinical explanations (42.0%, *n* = 126) (Fig. 3B).

Overall opinions toward AI in anatomy education were predominantly positive. 61.0% of students believed that AI as a helpful adjunct that should be used alongside traditional learning methods. A further 33.7% considered AI essential and supported its full integration into medical education. Only a small proportion perceived AI as having limited educational value (3.3%) or believed its use should be restricted or supervised (2.0%) (Fig. 3C).

Responses regarding the use of AI platforms as a complete standalone resource for studying anatomy reflected considerable doubt. Only 28.3% (*n* = 85) agreed (Strongly Agree + Agree), while 22.7% (*n* = 68) remained neutral. In contrast, 49.0% (*n* = 147) disagreed (Disagree + Strongly Disagree), suggesting that most students view AI as a complementary tool rather than a replacement for traditional learning approaches (Fig. 3D).

### Demographic comparisons of survey responses

A series of chi-square tests of independence were conducted to examine associations between gender and responses, as well as between academic year (2nd vs. 3rd year) and responses, across 20 survey questions regarding using AI platforms in anatomy education.

Before adjustment for multiple comparisons, statistically significant associations with gender were observed for two questions (Table 3). First, a significant association was found for the statement “AI platforms could replace some aspects of traditional teaching” (*p* = 0.007). Male students demonstrated higher rates of agreement, whereas female students were more likely to report a neutral response and exhibited lower levels of agreement. Second, a significant association was observed for “AI platforms sometimes provide complex or unclear explanations” (*p* = 0.023). Male students were more likely to disagree with this statement, while female students were more likely to agree that AI platforms provide complex or unclear explanations. However, after applying the Benjamini–Hochberg false discovery rate (FDR) correction for multiple comparisons, neither association remained statistically significant (BH-adjusted *p* = 0.140 and 0.230, respectively).

For the remaining 18 survey items, no statistically significant associations with gender were identified (all raw *p* > 0.05). Additionally, no statistically significant associations were observed between academic year (2nd vs. 3rd year) and responses for any of the 20 survey questions (all raw *p* > 0.05).

**Table 3:** Comparison of students’ perceptions, challenges, future perspectives, and overall opinions regarding the use of AI platforms in anatomy learning according to gender and academic year. Data are presented as frequency of responses. Associations were analyzed using the chi-square test, and effect sizes are reported as Cramér’s V. Raw p-values and Benjamini–Hochberg false discovery rate (FDR)-adjusted p-values are presented separately for gender and academic year comparisons to account for multiple testing. Statistical significance was defined as an FDR-adjusted *p* < 0.05. * For comparisons in which the assumptions of the chi-square test were not met, raw p-values were obtained using Fisher–Freeman–Halton exact test

Statement	Response	Male	Female	Raw *p*-value	Effect Size	FDR adjusted *p*-value	2nd Year	3rd Year	Raw *p*-value	Effect Size	FDR adjusted *p*-value
AI platforms help me understand and retain anatomical concepts	Agree	102	172	1.000*	0.015	1.000	133	141	0.256*	0.100	0.564
	Neutral	8	15				7	16			
	Disagree	1	2				1	2			
AI platforms provide immediate answers to my anatomy-related questions	Agree	101	166	0.406*	0.091	0.690	130	137	0.104*	0.127	0.480
	Neutral	10	19				11	18			
	Disagree	0	4				0	4			
AI offers personalized learning, including native language support	Agree	91	159	0.681	0.051	0.884	118	132	0.520	0.066	0.712
	Neutral	17	23				20	20			
	Disagree	3	7				3	7			
AI platforms are available 24/7, making them convenient	Agree	101	165	0.401	0.078	0.690	124	142	0.509	0.067	0.712
	Neutral	8	15				10	13			
	Disagree	2	9				7	4			
AI platforms help me prepare better for anatomy examinations	Agree	93	157	0.971	0.014	1.000	123	127	0.250*	0.099	0.564
	Neutral	15	26				15	26			
	Disagree	3	6				3	6			
AI platforms enhance my critical thinking skills in anatomy	Agree	63	114	0.826	0.036	0.918	81	96	0.534	0.065	0.712
	Neutral	36	57				43	50			
	Disagree	12	18				17	13			
AI platforms help connect anatomical knowledge to clinical applications	Agree	81	152	0.326	0.086	0.690	102	131	0.105	0.123	0.480
	Neutral	25	31				32	24			
	Disagree	5	6				7	4			
AI platforms save time compared to traditional methods	Agree	99	159	0.055	0.139	0.367	127	131	0.144	0.114	0.480
	Neutral	6	25				11	20			
	Disagree	6	5				3	8			
AI platforms make learning more engaging and interesting	Agree	71	131	0.273	0.093	0.690	93	109	0.216	0.101	0.564
	Neutral	31	38				30	39			
	Disagree	9	20				18	11			
AI platforms improved overall academic performance	Agree	85	142	0.792	0.039	0.918	111	116	0.310	0.088	0.564
	Neutral	23	39				27	35			
	Disagree	3	8				3	8			
AI lacks real visual and lab experience	Agree	67	132	0.122	0.118	0.552	95	104	0.870	0.030	0.899
	Neutral	22	35				25	32			
	Disagree	22	22				21	23			
AI platforms sometimes provide complex or unclear explanations	Agree	49	103	0.023	0.158	0.230	76	76	0.136	0.115	0.480
	Neutral	29	55				42	42			
	Disagree	33	31				23	41			
The cost of premium AI platforms is a barrier	Agree	64	122	0.138	0.115	0.552	85	101	0.837	0.034	0.899
	Neutral	24	44				33	35			
	Disagree	23	23				23	23			
AI platforms lack personal interaction and guidance	Agree	55	103	0.414	0.077	0.690	80	78	0.387	0.080	0.645
	Neutral	31	55				38	48			
	Disagree	25	31				23	33			
AI platforms do not always understand user questions	Agree	63	111	0.707	0.048	0.884	87	87	0.063	0.136	0.480
	Neutral	25	46				36	35			
	Disagree	23	32				18	37			
I plan to continue using AI platforms	Agree	101	175	0.320*	0.089	0.690	132	144	0.695*	0.058	0.842
	Neutral	6	12				7	11			
	Disagree	4	2				2	4			
I would recommend AI platforms to others	Agree	100	170	0.564*	0.066	0.806	128	142	0.899*	0.032	0.899
	Neutral	10	14				10	14			
	Disagree	1	5				3	3			
AI platforms should be formally integrated into the curriculum	Agree	89	137	0.326	0.086	0.690	105	121	0.140	0.114	0.480
	Neutral	16	37				22	31			
	Disagree	6	15				14	7			
AI platforms could replace some aspects of traditional teaching	Agree	71	86	0.007	0.182	0.140	69	88	0.295	0.090	0.564
	Neutral	20	58				36	42			
	Disagree	20	45				36	29			
AI platforms could serve as a complete standalone resource for studying anatomy	Agree	34	51	0.473	0.071	0.728	41	44	0.716	0.047	0.842
	Neutral	21	47				29	39			
	Disagree	56	91				71	76			

### Correlation between GPA and different domains of AI perceptions in anatomy learning

Self-reported GPA was selected for correlation analysis to assess whether perceptions of AI platforms are associated with students’ educational achievement. Overall, most questionnaire items demonstrated no significant correlation with GPA (*p* > 0.05), indicating that students’ academic performance was generally not associated with their perceptions of the benefits or limitations of AI platforms (Table 4).

**Table 4:** Correlation between students’ GPA and their perceptions, challenges, future perspectives, and overall opinions regarding the use of AI platforms in anatomy learning. Correlations were analyzed using Spearman’s rank correlation coefficient (rho). The table presents Spearman’s rho, 95% confidence intervals (95% CI), raw p-values, and Benjamini–Hochberg false discovery rate (FDR)-adjusted p-values. Statistical significance was defined as an FDR-adjusted *p* < 0.05

Statement	GPA		
	rho (95% CI)	Raw *p*-value	FDR adjusted *p*-value
Perceived Benefits of AI Platforms in Anatomy Learning			
AI platforms help me understand and retain anatomical concepts	0.078 (-0.039–0.193)	0.176	0.471
AI platforms provide immediate answers to my anatomy-related questions	< 0.000 (-0.116–0.117)	0.998	0.998
AI offers personalized learning, including native language support	0.025 (-0.092–0.141)	0.667	0.889
AI platforms are available 24/7, making them convenient	0.135 (0.018–0.247)	0.020	0.133
AI platforms help me prepare better for anatomy examinations	0.093 (-0.024–0.207)	0.109	0.363
AI platforms enhance my critical thinking skills in anatomy	-0.002 (-0.119–0.114)	0.969	0.998
AI platforms help connect anatomical knowledge to clinical applications	0.010 (-0.106–0.127)	0.857	0.952
AI platforms save time compared to traditional methods	0.073 (-0.044–0.188)	0.209	0.471
AI platforms make learning more engaging and interesting	-0.033 (-0.149–0.084)	0.575	0.821
AI platforms improved overall academic performance	0.055 (-0.062–0.170)	0.345	0.575
Challenges and Limitations of AI Platforms in Anatomy Learning			
AI lacks real visual and lab experience	0.072 (-0.045–0.187)	0.212	0.471
AI platforms sometimes provide complex or unclear explanations	-0.068 (-0.183–0.049)	0.243	0.482
The cost of premium AI platforms is a barrier	0.016 (-0.101–0.132)	0.788	0.952
AI platforms lack personal interaction and guidance	0.065 (-0.052–0.180)	0.265	0.482
AI platforms do not always understand user questions	-0.012 (-0.128–0.105)	0.837	0.952
Future perspective			
I plan to continue using AI platforms	0.112 (-0.005–0.225)	0.054	0.216
I would recommend AI platforms to others	0.117 (0.001–0.230)	0.043	0.215
AI platforms should be formally integrated into the curriculum	0.047 (-0.070–0.163)	0.415	0.638
AI platforms could replace some aspects of traditional teaching	-0.143 (-0.256 – -0.027)	0.013	0.130
Overall Opinion			
AI platforms could serve as a complete standalone resource for studying anatomy	-0.184 (-0.295 – -0.069)	0.001	0.020

However, before adjustment for multiple comparisons, some statistically significant associations were identified. A weak significantly positive correlation was observed between GPA and the perception that AI platforms are available 24/7 and convenient for learning (rho = 0.135, *p* = 0.020) and recommending AI platforms to others (rho = 0.117, *p* = 0.043). In contrast, significant negative correlations were found between GPA and the perception that AI platforms could replace some aspects of traditional teaching (rho = -0.143, *p* = 0.013), as well as the belief that AI platforms could serve as a complete standalone resource for studying anatomy (rho = -0.184, *p* = 0.001).

After applying the Benjamini–Hochberg false discovery rate (FDR) correction for multiple comparisons, only the negative correlation between GPA and the belief that AI platforms could serve as a complete standalone resource for studying anatomy remained statistically significant (adjusted *p* = 0.020). These findings may suggest that students with higher academic performance tend to view AI platforms as supportive learning tools rather than complete substitutes for traditional anatomy education.

## Discussion

This study provides a comprehensive assessment of AI use in anatomy learning among medical students. The findings show that AI use was commonly reported among respondents, with most indicating that they used AI to support their anatomy learning. Students viewed AI primarily as a learning adjunct, strongly endorsing its benefits for conceptual understanding, immediate availability, and exam preparation. However, they expressed important concerns about the lack of visual/laboratory experience, cost, and AI’s occasional misinterpretation of questions. Interestingly, students with higher GPAs were less likely to view AI as a replacement for traditional teaching and more likely to value it as a convenient adjunct. Although male students showed greater agreement than female students that AI could replace aspects of conventional instruction in the unadjusted analysis, this association did not remain statistically significant after FDR correction. Overall, the findings suggest that AI use was common among respondents and was accompanied by generally favorable but cautious perceptions of its role in anatomy education.

The high proportion of respondents reporting AI use is consistent with findings from recent international cross-sectional studies. For instance, in a U.S. cross-sectional study, medical students widely used ChatGPT for understanding complex concepts, generating study materials, and exam preparation [16]. Similarly, in a global multi-country survey covering medical, dental, and veterinary students, overall positive perceptions of AI use in healthcare and education predominated [17]. In the Middle East, a Saudi study reported broadly favorable AI attitudes but with notable knowledge gaps [18]. Likewise, an Iraqi study found high acceptance but limited formal training [19]. ChatGPT emerged as the most frequently used AI platform among participants in our study. This observation is consistent with the broader medical education literature, which has identified accessibility, ease of use, and free availability as features associated with students’ preference for ChatGPT [8, 16]. Moreover, nearly half of the participants reported more than one year of AI experience, which is consistent with sustained use of AI in anatomy learning among many participants. Furthermore, gross anatomy was the most frequently consulted topic, which may reflect the discipline’s dense terminology and conceptual complexity. Such content may be particularly amenable to the explanatory and interactive functions of large language models (LLMs) [20, 21]. Collectively, these findings are consistent with students’ use of AI as a supplementary resource in anatomy education and self-directed learning.

The perceived benefits observed in our study aligned with the known educational capabilities of LLMs. Specifically, the highest rated item, “AI supports the understanding and retention of anatomical concepts,” is consistent with evidence that AI can synthesize and simplify complex anatomical content [20]. Additionally, high agreement that AI provides immediate answers and 24/7 availability reflects another well-documented advantage that may help mitigate limitations associated with faculty availability and access to learning resources [8, 22]. Similarly, time-saving, personalized learning, and improved exam preparation were also positively perceived benefits, consistent with international surveys reporting that students value AI’s adaptive, individualized responses [8, 20]. Moreover, students perceived AI as useful for connecting anatomy to clinical scenarios, which is particularly important in modern integrated curricula where vertical integration of basic sciences with clinical reasoning is a pedagogical priority [22, 23].

Regarding the challenges and limitations, the most prominent concern was the lack of visual/laboratory experience. Cadaveric dissection, plastinated specimens, and physical models offer kinesthetic and spatial learning experiences that AI text-based learning tools cannot replicate [2, 24]. This finding suggests many students recognize that AI should complement rather than replace the hands-on experiences that remain fundamental to anatomy education. Furthermore, this perception is congruent with reviews of digital anatomy modalities, which conclude that 3D, VR, and AR tools can supplement but not fully replace cadaver-based instruction [2, 25]. The cost of premium platforms has practical equity implications. Although free-tier LLMs are widely available, more capable models require paid subscriptions that may be beyond the reach of many students, particularly in resource-constrained settings. This barrier raises concerns about a digital divide in AI-augmented learning [26]. Students also reported concerns that AI may misunderstand their questions or provide complex or unclear explanations. These perceptions may be consistent with documented limitations of LLMs, including hallucinations, and may also relate to variation in prompt construction and response quality [20, 27]. Indeed, recent evaluations of ChatGPT’s anatomy performance have reported varying accuracy rates ranging from moderate to high, with notable low performance in spatial relationships, neuroanatomy, and embryological detail [20, 21]. A notable finding was that more than one-third of students reported not routinely verifying AI-generated information. Given the documented risk of hallucinations and factual inaccuracies in LLM outputs on medical topics [20, 27], this finding may indicate a potential educational vulnerability and supports consideration of structured AI literacy training, including critical evaluation and prompting skills [28, 29].

The majority of students supported the formal integration of AI into the anatomy curriculum, mirroring international trends in which students consistently call for structured AI training [17, 28, 30, 31]. However, only about half believed AI could replace some aspects of traditional teaching. Additionally, nearly half of the respondent students disagreed that AI could serve as a complete standalone resource for studying anatomy. Taken together, these responses are consistent with support for an integrated hybrid model in which AI supplements, rather than replaces, cadaveric dissection, lectures, and faculty-led teaching. This interpretation is consistent with calls in the literature for blended curricular models that combine traditional methods, digital anatomy tools, and AI-assisted learning [2, 24, 25]. Although most students reported being somewhat or very confident in using AI for anatomy, competence in detecting AI errors was not objectively assessed. Accordingly, reported confidence should not be interpreted as evidence of such competence. Together with prior literature, these findings support consideration of explicit instruction in AI literacy, ethical use, and academic integrity [28, 29].

Although two gender-related trends were observed in the unadjusted analyses, these associations were no longer statistically significant after Benjamini–Hochberg false discovery rate correction. Male students showed a greater tendency than female students to agree that AI could replace aspects of traditional teaching, and female students were more likely to view AI-generated explanations as complex or unclear. Although these unadjusted patterns resemble findings reported in some previous studies [30, 32, 33], they should be considered exploratory and require replication in larger studies before potential explanatory factors are proposed. Interestingly, after FDR correction, the only statistically significant GPA-related finding was a negative association between GPA and agreement that AI could serve as a complete standalone resource for studying anatomy. This association is consistent with students with higher GPA being less likely to regard AI as a complete substitute for traditional learning. However, the present study did not assess actual AI-use strategies, metacognitive skills, or AI dependence. Conversely, lower GPA was associated with greater agreement that AI could serve as a standalone resource, but whether this attitude corresponds to greater reliance on AI or is associated with differences in knowledge consolidation, clinical reasoning, or long-term retention requires longitudinal investigation [34–36].

### Limitations

Several limitations must be acknowledged. First, the cross‑sectional design captures perceptions at one time point and cannot infer causality or track evolving attitudes. Second, the single‑center setting (JUST) limits generalizability, so multicenter studies are needed. Third, self‑reported data are susceptible to recall, social desirability, and self-selection biases, possibly over-representing AI‑enthusiastic students. In addition, GPA was not verified through academic records, which may have introduced reporting bias. Fourth, we did not objectively assess the accuracy or educational impact of AI‑supported study; only subjective perceptions were assessed, so future research should link AI use to objective outcomes, such as exam performance. Moreover, although the achieved sample size met the a priori sample size requirement, the response rate was 27.0%, which may have introduced self-selection bias. Students with greater interest in AI or digital learning may have been more likely to participate, potentially limiting the generalizability of the findings to the overall medical student population. Finally, the rapidly evolving AI landscape means results may quickly become dated, requiring longitudinal follow‑up.

## Conclusion

Among 300 medical students from JUST who use AI in their anatomy learning, most students reported using AI as a valuable adjunct in their anatomy learning, praising its accessibility, conceptual support, and exam‑preparation utility. Despite these benefits, important limitations have been recognized, particularly the inability of AI to replace hands‑on laboratory experiences and concerns about the accuracy of AI‑generated information. Attitudes toward AI in anatomy learning varied according to sex and academic performance, with higher‑achieving students being more likely to perceive AI as a supportive tool rather than a substitute for traditional instruction. The finding that a substantial proportion of respondents do not verify AI‑generated information highlights the need for structured AI literacy training. Overall, these findings support the integration of AI into anatomy education as a complementary educational resource within a balanced, hybrid learning model that preserves the essential role of faculty guidance and practical anatomical training.

## Supplementary Information

Supplementary Material 1.

## Data Availability

All data supporting the findings of this study are available within the paper and its Supplementary Information.
