# Supplementary material for: Perceptions and attitudes of medical students toward AI in anatomy learning
Source: BMC Med Educ. 2026 Aug 3;26:1751. doi: 10.1186/s12909-026-10085-1 (PMC13721423; doi:10.1186/s12909-026-10085-1)
Supplement: Supplementary file 1 [file 12909_2026_10085_MOESM1_ESM.pdf]

Perceptions and Attitudes of Medical Students Toward AI in Anatomy Learning

* Indicates required question

1. Dear Medical Student, *

We are recently conducting a research survey on the perceptions and attitudes of medical students toward AI in anatomy learning, and we are kindly requesting your participation in this study.

This study aims to explore the perceptions, attitudes, and perceived challenges of medical students in using AI in learning human anatomy. Our ultimate goals are to explore the perceptions, attitudes, and challenges faced by medical students regarding the use of Artificial Intelligence platforms in learning human anatomy. Therefore, your participation by providing us with your viewpoint is very important in achieving more comprehensive and more relevant feedback about your experience in using AI in learning human anatomy.

The study involves completing an **anonymous** questionnaire about using AI in learning human anatomy. Participation is entirely **voluntary**, and you **may withdraw** from the study at any time before completing the questionnaire by simply closing the webpage or exiting the link. The study is completely anonymous; therefore, it does not require you to provide your name, student ID, or other identifying information. By agreeing to participate in the study, you will be **giving your consent** for the researchers to include your responses in their data analysis. There will be no individually identifiable information, remarks, comments, or other identification of you as an individual student participant. All results will be presented as aggregate, summary data.

The survey should last no more than 5-10 minutes. Your participation in the research will be of great importance to gain a deeper understanding of the perceptions and attitudes of our medical students toward the subject of AI in human anatomy.

Ethical approval **was obtained** from the Research Ethics Committee at JUST. We look forward to your participation in this study. Please do not hesitate to contact the principal investigators (Dr. Ibrahim Hoja) if you have any questions through the email address: hoja_78@just.edu.jo

I agree to participate in this study

Mark only one oval.

- Yes
- No *Skip to section 9 (END OF QUESTIONNAIRE.)*

SECTION A: DEMOGRAPHIC INFORMATION

2. What is your age (year)? *

Mark only one oval.

- 18
- 19
- 20
- 21
- 22
- 23
- ≥ 24

3. Gender *

Mark only one oval.

- Male
- Female

4. Current year of medical school *

Mark only one oval.

- 2nd year
- 3rd year

5. Overall Grade Point Average (GPA): *

Mark only one oval.

- Below 2.0 (On probation)
- 2.00 – 2.49 (Fair)
- 2.50 - 2.99 (Good)
- 3.00 - 3.49 (Very Good)
- 3.50 – 3.99 (Excellent)
- Above 4.00 (Distinguished)

6. What is your program enrollment type? *

Mark only one oval.

- Regular
- Parallel
- International

7. Do you currently have access to any AI platforms? *

Mark only one oval.

- Yes, free versions
- Yes, paid/premium versions

SECTION B: USAGE PATTERNS OF AI PLATFORMS FOR ANATOMY LEARNING

8. How long have you been using AI platforms for **anatomy learning**? *

Mark only one oval.

- I don't use AI for Anatomy learning
- Less than 6 month
- 6-12 months
- More than 1 year

9. Which AI platform(s) do you use most **COMMONLY** for anatomy learning? (Select **ONE** or **TWO** choices) *

Check all that apply.

- [ ] ChatGPT
- [ ] Claude
- [ ] Google Gemini (Bard)
- [ ] DeepSeek
- [ ] Grok
- [ ] Genspark
- [ ] Perplexity AI
- [ ] Microsoft Copilot

10. How do you **primarily** use AI platforms for anatomy learning? (Select **ONE** or **TWO** Choices) *

Check all that apply.

- [ ] Asking questions about anatomical structures
- [ ] Requesting explanations of complex concepts
- [ ] Generating study materials (summaries, flashcards)
- [ ] Understanding clinical correlations
- [ ] Understanding spatial relationships (Link anatomy text to visual representations)
- [ ] Preparing for exams (practice questions)

11. How frequently do you use AI platforms for anatomy learning? *

Mark only one oval.

- For most anatomy sessions
- For several anatomy sessions
- For a few sessions

12. When do you typically use AI for anatomy learning? (Select all that apply) *

Check all that apply.

- [ ] Before class
- [ ] During class or lab
- [ ] While reviewing or studying for exams
- [ ] After an exam to understand mistakes

13. What is/are the most anatomical topics you use AI platforms for? (Select **ONE or TWO** options only) *

Check all that apply.

- [ ] Gross anatomy
- [ ] Neuroanatomy
- [ ] Embryology
- [ ] Histology
- [ ] Radiological anatomy
- [ ] Clinical anatomy
- [ ] Surface anatomy

14. Which traditional learning resources do you combine with AI platforms? (Select all that apply) *

Check all that apply.

- [ ] Textbooks and atlases
- [ ] Video lectures (e.g., YouTube)
- [ ] 3D anatomy software
- [ ] Cadaver Prosection
- [ ] Anatomy models
- [ ] Lecture notes & sheets

SECTION C: PERCEIVED BENEFITS OF AI PLATFORMS

15. Please provide your opinion on the following statements: *

Mark only one oval per row.

	Strongly Agree	Agree	Neutral	Disagree	Strongly Disagree
**AI platforms help me understand and retain anatomical concepts**					
**AI platforms provide immediate answers to my anatomy-related questions**					
**AI offer personalized learning, including native-language support for complex topics**					
**AI platforms are available 24/7, making them convenient for studying**					
**AI platforms help me prepare better for anatomy examinations**					
**AI platforms enhance my critical thinking skills in anatomy**					
**AI platforms help me connect anatomical knowledge to clinical applications**					
**AI platforms save me time compared to traditional learning methods**					
**AI platforms make anatomy learning more engaging and interesting**					
**AI platforms have improved my overall academic performance in anatomy**					

SECTION D: CHALLENGES AND LIMITATIONS

16. To what extent do you agree with the following challenges of AI platforms for anatomy learning? *

Mark only one oval per row.

	Strongly Agree	Agree	Neutral	Disagree	Strongly Disagree
**AI lacks real visual and lab experience**					
**AI platforms sometimes provide complex or unclear explanations**					
**The cost of premium AI platforms is a barrier to their use**					
**AI platforms lack the personal interaction and guidance of human instructors**					
**AI platforms do not always understand what I am asking**					

SECTION E: VERIFICATION AND TRUST

17. How do you verify the accuracy of information provided by AI platforms? (Select all that apply) *

Check all that apply.

- [ ] I don't usually verify the information
- [ ] Cross-reference with textbooks and atlases
- [ ] Consult with instructors and professors
- [ ] Compare with other AI platforms and/or online resources
- [ ] Discuss with my peers and classmates
- [ ] Ask the AI to generate answers from reliable resources (textbooks or published articles)

18. How much do you trust the anatomical information provided by AI platforms? *

Mark only one oval.

- Completely
- Mostly
- Somewhat
- Rarely
- Do not trust at all

19. Have you ever received incorrect information from an AI platform regarding anatomy? *

Mark only one oval.

- Yes, frequently
- Yes, occasionally
- Yes, rarely
- No, never
- Not sure/Can't remember

20. Compared to traditional learning methods (textbooks, lectures, dissection), how effective do you find AI platforms for anatomy learning? *

Mark only one oval.

- Much more effective
- Somewhat more effective
- Equally effective
- Somewhat less effective
- Much less effective

SECTION F: FUTURE PERSPECTIVES

21. To what extent do you agree with the following statements about the future of AI in anatomy learning? *

Mark only one oval per row.

	Strongly Agree	Agree	Neutral	Disagree	Strongly Disagree
**I plan to continue using AI platforms**					
**I would recommend AI platforms to other**					
**AI platforms should be formally integrated into the curriculum**					
**AI platforms could eventually replace some aspects of traditional anatomy teaching**					

Skip to question 22

SECTION G: CONCLUSION SECTION

22. How confident are you in your ability to use AI tools effectively for learning anatomy? *

Mark only one oval.

- Very confident
- Somewhat confident
- Neutral
- Slightly unconfident
- Not confident at all

23. Which features, or improvements would make AI platforms more useful for anatomy learning? (Select all that apply) *

Check all that apply.

- [ ] Integration with anatomy models, pictures, or 3D apps
- [ ] More accurate medical information
- [ ] Better clinical explanations
- [ ] Customization to match my university's curriculum

24. Your overall opinion about AI in anatomy learning: *

Mark only one oval.

- AI is essential and should be fully integrated into medical education
- AI is helpful, but should be used with traditional methods
- AI has limited educational value
- AI use should be restricted or supervised

25. Overall, can AI platforms serve as a complete (standalone) resource for studying anatomy: *

Mark only one oval.

- Strongly Agree
- Agree
- Neutral
- Disagree
- Strongly Disagree

END OF QUESTIONNAIRE.

Thank You very much

This content is neither created nor endorsed by Google.

Google Forms
